# An Introduction to the Study and Practice of Medicine

**Published:** 1834-04-01

**Authors:** 


*An Introduction to the Study and Practice of Medicine.* By JOHN DOWSON, M.D. London, 1834. 12mo. pp. 96.

THIS is a very harmless little book, probably intended for distribution among the author's circle of friends: should he reprint it, we would recommend him to enlarge it considerably, as ninety-six very tiny pages on such a subject leave no room for doing justice even to the most important points. The following passage, which our author quotes from Parkinson, is a curious example of a commonplace truth so distorted by exaggeration that the fact can scarcely be recognised amid the embellishments.

"You must have observed how late it is before a physician gets into full practice; and that few of them begin to derive any advantage from their profession until they have arrived at that time of life at which men engaged in business, or mercantile concerns, have generally acquired a handsome fortune. Indeed it almost seems that many think a physician too young for consultation, unless he has reached his dotage: I have myself known a physician, above fifty years of age, objected to for his youth." (P. 64.)

These objectors to a younker of fifty must be very hard to please: they would allow, perhaps, that a physician who had numbered threescore years and ten might be trusted with slight cases, but they would hardly give their full confidence to any but a Heberden in his ninetieth year, or a Glisson in his hundredth. We suspect, however, that the unfortunate quinquagenarian in the text must have been afflicted with a sleek and chubby face; though it is strange that the atrophy of the purse, the entire and absolute unconsciousness of a fee, which Mr. Parkinson affixes on our younger brethren, should not have planted a few wrinkles on the halest cheek. But some men are born stoics.

